# An Anatomical Site and Genetic-Based Prognostic Model for Patients With Nuclear Protein in Testis (NUT) Midline Carcinoma: Analysis of 124 Patients

**DOI:** 10.1093/jncics/pkz094

**Published:** 2019-11-06

**Authors:** Nicole G Chau, Clement Ma, Kristina Danga, Hasan Al-Sayegh, Valentina Nardi, Ryan Barrette, Christopher S Lathan, Steven G DuBois, Robert I Haddad, Geoffrey I Shapiro, Stephen E Sallan, Arindam Dhar, Jeanenne J Nelson, Christopher A French

**Affiliations:** 1 Dana-Farber Cancer Institute, Boston, MA; 2 Harvard Medical School, Boston, MA; 3 Dana-Farber/Boston Children’s Cancer and Blood Disorders Center, Boston, MA; 4 Brigham and Women’s Hospital, Boston, MA; 5 Massachusetts General Hospital, Boston, MA; 6 GlaxoSmithKline, Collegeville, PA

## Abstract

**Background:**

NUT midline carcinoma, renamed NUT carcinoma (NC), is an aggressive squamous cancer defined by rearrangement of the *NUTM1* gene. Although a subset of patients can be cured, for the majority of patients the prognosis is grim. We sought to classify patients into risk groups based on molecular and clinicopathologic factors at the time of diagnosis.

**Methods:**

Clinicopathologic variables and survival outcomes were extracted for a total of 141 NC patients from the NUT midline carcinoma Registry using questionnaires and medical records. Translocation type was identified by molecular analyses. Survival tree regression analysis was performed to determine risk factors associated with overall survival (OS).

**Results:**

For 141 patients, the median age at diagnosis was 23.6 years. Fifty-one percent had thoracic origin compared with 49% nonthoracic sites (41% head and neck, 6% bone or soft tissue, 1% other). The median OS was 6.5 months (95% confidence interval [CI] = 5.8 to 9.1 months). Most patients had the *BRD4-NUTM1* fusion (78%), followed by *BRD3-NUTM1* (15%) and *NSD3-NUTM1* (6%). Survival tree regression identified three statistically distinct risk groups among 124 patients classified by anatomical site and genetics: group A is nonthoracic primary, BRD3-, or NSD3-NUT (n* = *12, median OS = 36.5 months, 95% CI = 12.5 to not reported months); group B is nonthoracic primary, BRD4-NUT (n* = *45, median OS = 10 months, 95% CI = 7 to 14.6 months); and group C is thoracic primary (n* = *67, median OS = 4.4 months, 95% CI = 3.5 to 5.6 months). Only groups A and B had long-term (≥3 years, n = 12) survivors.

**Conclusions:**

We identify three risk groups defined by anatomic site and *NUT* fusion type. Nonthoracic primary with non-*BRD4-NUT* fusion confers the best prognosis, followed by nonthoracic primary with *BRD4-NUT*. Thoracic NC patients, regardless of the *NUT* fusion, have the worst survival.

Chromosomal rearrangement of the Nuclear protein in testis (*NUTM1*, aka *NUT*) gene defines a rare subtype of squamous cell carcinoma termed NUT carcinoma (NC, also known as NUT midline carcinoma [NMC]) (1,2). NC is important to recognize because it is clinically distinct from other carcinomas. With a median overall survival (OS) of 6.7 months, it is possibly the most aggressive solid tumor with the worst prognosis in humans (3). In NC, *NUTM1* is most often fused to Bromodomain-containing protein 4 (*BRD4*) forming a *BRD4-NUTM1* fusion oncogene but can also be fused to a variety of other partner genes, including Bromodomain-containing protein 3 (*BRD3*), Nuclear Receptor Binding SET Domain Protein 3 (*NSD3*), Zinc Finger Protein 532 (*ZNF532*), Zinc Finger Protein 592 (*ZNF592*), Capicua transcriptional repressor (*CIC*), and other yet unidentified genes (1,3–9). The aggressiveness of NC is attributable to the NUT fusion oncoproteins that drive its growth by upregulation of MYC (6,10,11). In this report, we refer to the gene as *NUTM1* and the encoded protein as NUT. Patients are of all ages but most commonly are in their early twenties (12).

Although NC is aggressive, we have noted that outcomes and response to therapy can be quite variable (3,12–14). Despite the heterogenous outcomes in NC, its underdiagnosis and rarity have thus far precluded the ability to identify favorable vs unfavorable groups. Having accumulated the largest existing cohort of NC patients with molecular and clinical data through the NMC Registry (www.NMCRegistry.org), we sought to develop a risk classification system for NC incorporating genetic and clinico-pathologic features in this study.

## Materials and Methods

### Patients

From January 1993 to July 2017, we identified a total of 141 NC patients from 17 countries registered in the NMC Registry (see Supplementary Methods and Supplementary Table 1, available online). Patients analyzed include those diagnosed before 2010 who were enrolled into the registry retrospectively (n = 63) and those enrolled prospectively from 2010 to July 2017 (n = 78). Ninety-two patients (65%) had previously been analyzed and reported by our group (3,12); however, this study provides additional clinical follow-up and *NUTM1* fusion partner identification for the majority (73%) of these and includes 49 additional NMC patients not previously reported. The diagnosis of NC was defined by rearrangement of *NUTM1* detected by one or more of the following methods: NUT immunohistochemistry (IHC) demonstrating more than 50% tumor nuclear staining (15–17), or *NUTM1* rearrangement by cytogenetics, fluorescent in situ hybridization (FISH), or next-generation sequencing (NGS)-based ArcherDx FusionPlex. The histology and NUT IHC for all cases was reviewed by one of our authors (C. A. French). Histology was categorized into three groups: 1) carcinoma without squamous differentiation, 2) carcinoma with squamous differentiation, or 3) other histopathology.

Patient clinical, demographic, treatment, and outcomes data were collected through questionnaires provided by treating physicians and chart review. Outcome data were obtained for 141 patients. Further details are in the Supplementary Material (available online).

### NUT IHC

IHC for NUT using primary rabbit monoclonal anti-NUT (clone C52B1, 1:50) is described in Supplementary Methods (available online).

### *NUTM1* Fusion Partner Gene Identification

*NUTM1* fusion type was determined by cytogenetics [as described (18)] using t(15; 19)(q14; p13.1) as evidence of *BRD4-NUTM1* fusion; FISH (see Supplementary Methods, available online), which tests for genomic fusions to *NUTM1* of candidate partner genes, *BRD4*, *BRD3*, *NSD3*, or *ZNF532*; or ArcherDx FusionPlex, a rapid amplification of cDNA ends-based NGS approach to identify any fusion partner to *NUTM1* (see Supplementary Methods, available online).

### Next-Generation (OncoPanel) Targeted Sequencing

OncoPanel is the in-house (Brigham and Women’s Hospital) targeted genomic NGS platform used to detect cancer-associated mutations and genomic rearrangements. Details of OncoPanel molecular profiling of formaldehyde fixed, paraffin-embedded (FFPE) sections are described in Supplementary Methods (available online). Exons of 447 cancer-associated genes were interrogated for mutations and copy number variations, and 191 introns across 60 genes were examined for structural rearrangements.

### Statistical Methods

Descriptive statistics were used to summarize patient demographic and clinical characteristics. OS was calculated from initial cancer diagnosis to death or to last follow-up if censored. Event-free survival (EFS) was calculated from initial cancer diagnosis to progression or death or to last follow-up if censored. Univariate Cox proportional-hazards regression was used to test the association of prognostic factors with OS and EFS in the full cohort. The proportional hazards assumption for univariate models were examined using log-log plots. Fisher exact test was used to compare patient clinical characteristics by primary tumor site.

We performed survival tree regression to create the risk classification model (19) as previously used in classifying patients with neuroblastoma (20). Survival tree regression can be more flexible than multivariable modeling because each branch of the tree can have a different number of subbranches with different risk factors. We considered the following risk factors for inclusion in the tree: age at diagnosis, sex, primary tumor site, tumor size, histopathologic type of tumor, translocation, lymph node or organ metastasis, and bone or soft tissue primary tumor. Starting with the full patient cohort, Cox proportional-hazards regression was performed for each potential risk factor. Patients were dichotomized into two subgroups using the risk factor with the lowest statistically significant (*P* < .05) *P* value. We repeated this process recursively within each subgroup until no further statistically significant factors were found or when a subgroup had fewer than 20 patients. We performed post hoc multivariable Cox proportional-hazards regression using the full cohort to examine potential confounders of selected risk factors in the risk classification model. Further details are in Supplemental Methods (available online). We conducted simulation studies to determine the minimum sample size of a prospective cohort of NC patients needed to validate the risk classification model. Further details are in Supplemental Methods (available online).

All analyses were performed in SAS version 9.4 (SAS Institute, Cary, NC). Two-sided *P* values less than or equal to .05 were considered statistically significant. All tests were two-sided.

## Results

### Patient Demographics and Tumor Characteristics

For 141 patients (Table 1), the median age at diagnosis was 23.6 years (range = 18 days–80 years) and 48% were male. Fifty-one percent (71 of 140) of tumors arose from the thorax, 41% (58 of 140) from head and neck, 6% (9 of 140) from bone and soft tissue, and 1% (2 of 140) from the kidney. As expected, the majority (99 of 127, 78%) of tumors harbored a *BRD4-NUTM1* fusion, whereas the second and third most prevalent fusion types were *BRD3-NUTM1* (19 of 127, 15%) and *NSD3-NUTM1* (7 of 127, 6%), respectively. Other fusions identified included *ZNF532-NUTM1* (6) and *ZNF592-NUTM1* (9). For 14 cases, the fusion partner gene to *NUTM1* was not identified because either it was not tested (n = 5) or it was tested by FISH but negative for the candidate genes tested (n = 9).

**Table 1.** Patient demographic, clinical, and tumor characteristics (N = 141)

Patient characteristic	No. (%) or median (range)
Age at initial cancer diagnosis, y	23.6 (18 d–80 y), n = 124
<18	47 of 124 (38)
≥18	77 of 124 (62)
Unknown	17
Sex	
Male	67 of 141 (48)
Female	74 of 141 (52)
Primary tumor site	
Thoracic	71 of 140 (51)
Head and neck	58 of 140 (41)
Bone and soft tissue*	9 of 140 (6)
Other site (kidney)	2 of 140 (1)
Unknown	1
Gene fusion	
*BRD4-NUTM1*	99 of 127 (78)
*BRD3-NUTM1*	19 of 127 (15)
*NSD3-NUTM1*	7 of 127 (6)
*ZNF532-NUTM1*	1 of 127 (1)
*ZNF592-NUTM1*	1 of 127 (1)
Unknown NUTM1 (fusion partner was tested, but not identified)	9
Not tested	5
Histological type	
Carcinoma with squamous differentiation	46 of 138 (33)
Carcinoma without squamous differentiation	75 of 138 (54)
Other histology	17 of 138 (12)
Unknown	3
Tumor diameter at diagnosis, cm	5.6 (0.4–16.2), n = 86
Tumor diameter at diagnosis	
<6 cm	46 of 86 (53)
≥6 cm	40 of 86 (47)
Unknown	55
Lymph node and/or organ metastasis at presentation	
Yes	71 of 113 (63)
No	42 of 113 (37)
Unknown	28
Initial treatment sequence	
Chemotherapy ± subsequent treatment	41 of 112 (37)
Radiation ± concurrent chemotherapy ± subsequent treatment	31 of 112 (28)
Surgery ± subsequent treatment	40 of 112 (36)
No treatment or unknown	29
Initial response	
Complete or partial response	55 of 112 (49)
Stable or progressive disease	57 of 112 (51)
Unknown	29
Did patient ever receive radiation	
Yes	85 of 117 (73)
No	32 of 117 (27)
Unknown	24
Did patient ever receive chemotherapy	
Yes	105 of 117 (90)
No	12 of 117 (10)
Unknown	24
Did patient ever receive surgery	
Yes	60 of 118 (51)
No	58 of 118 (49)
Unknown	23
For N = 60 patients who ever received surgery	
Any microscopic margins involved (in any surgery)	
Yes	22 of 31 (71)
No	9 of 31 (29)
Unknown	29
Any gross residual disease postoperatively (in any surgery)	
Yes	31 of 47 (66)
No	16 of 47 (34)
Unknown	13

* Bone and soft tissue primary tumors include chest wall soft tissue, soft tissue mass, right hip bone, left scapula or shoulder, right chest soft tissue, soft tissue, occipital scalp soft tissue, iliac bone, and temporal region soft tissue mass.

The most common histology was carcinoma without squamous differentiation (75 of 138 cases, 54%); however, morphologic squamous differentiation, as evidenced by focal squamous “pearls” or stratification or enlargement of tumor cells, was seen in 33% (46 of 138) of cases. Tumors lacking evidence of epithelial differentiation or where histologic classification was not specified composed the remaining (17 of 138, 12%) cases. The median tumor size (diameter) at diagnosis was 5.6 cm (range = 0.4–16.2 cm, n* = *86), and the majority of patients presented with lymph node and/or organ metastases (71 of 113, 63%).

### Outcomes

**Figure 1.**
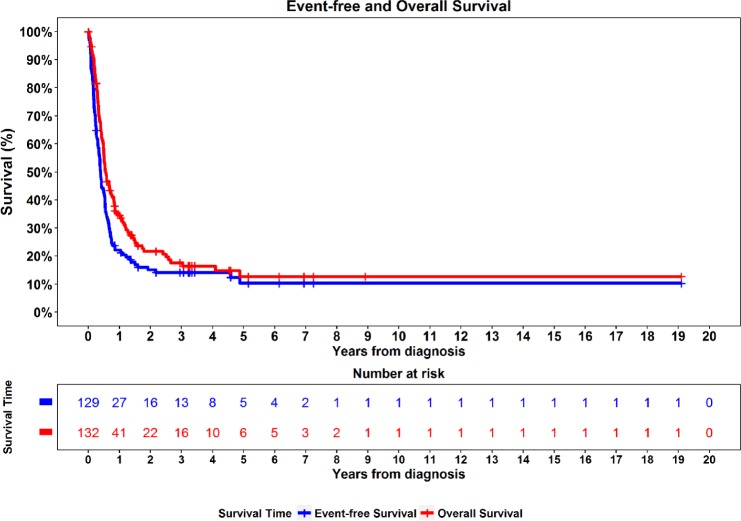
Kaplan-Meier curves for overall survival (N = 132) and event-free survival (N = 129).

Outcomes in NC patients were overall poor and consistent with previous data (3,12) (Figure 1). Median follow-up was 2.9 years (1 day–19.1 years) among 27 patients alive at last contact. Median OS and EFS were 6.5 months (95% confidence interval [CI] = 5.8 to 9.1 months) and 4.6 months (95% CI = 3.8 to 6.2 months), respectively. Two-year OS and EFS were only 22% (95% CI = 15% to 30%) and 15% (95% CI = 9% to 22%), respectively. Nevertheless, there were 16 long-term survivors, defined as living at least 3 years. Of these, only 12 patients had sufficient data to analyze for the risk stratification model (see below).

Response to initial treatment was also generally poor with only 49% (55 of 112) of patients with complete or partial response to initial therapy. The majority of patients received multimodality therapy (90 of 117, 78%).

### Prognostic Risk Factors for Survival Outcomes

Univariate Cox regression analysis of prognostic factors revealed the novel finding that translocation type correlates statistically significantly with prognosis (Table 2). Of 117 patients, the presence of *BRD4-NUTM1* fusions conferred a poorer OS than those with *BRD3-NUTM1* or *NSD3-NUTM1* (hazard ratio [HR] = 1.8, 95% CI = 1.1 to 3.1, *P = *.024). Consistent with previous observations (3,12), thoracic primary tumor site vs “nonthoracic” sites (head and neck, bone, soft tissue, and other sites; *P* < .0001), initial presentation with lymph node or organ metastases (*P = *.01), and lack of initial complete response or partial response (*P* < .001) to treatment was statistically significantly associated with worse OS and EFS (Table 2). Larger tumor size of 6 cm or greater was associated with statistically significantly worse EFS (*P* = .029). The observation that treatment with surgery or radiation (at any time point) is associated with improved OS (*P* < .0001) and EFS (*P* < .004) was confirmed in this study.

**Table 2.** Univariate Cox proportional-hazards regression of prognostic factors for OS and EFS*

			OS			EFS		
Factor	Level	Reference	No.	HR (95% CI)	*P*	No.	HR (95% CI)	*P*
Age at diagnosis, y	≥18	<18	121	1.5 (1 to 2.3)	.06	118	1.3 (0.9 to 2)	.2
Sex	Female	Male	132	1 (0.7 to 1.4)	.8	129	1 (0.7 to 1.5)	.8
Primary tumor site	Thoracic	Nonthoracic (head and neck, bone, soft tissue, and other)	131	3.4 (2.2 to 5.1)	<.0001	128	3.2 (2.2 to 4.9)	<.0001
Tumor size, cm	≥6	<6	83	1.5 (0.9 to 2.4)	.1	81	1.7 (1.1 to 2.7)	.029
Histopathologic type of tumor	Carcinoma without squamous differentiation and other histopathology	Carcinoma with squamous differentiation	129	1.2 (0.8 to 1.8)	.4	126	1.2 (0.8 to 1.8)	.4
Gene fusion	BRD4-NUTM1	BRD3-NUTM1 or NSD3-NUTM1	117	1.8 (1.1 to 3.1)	.024	114	1.7 (1 to 2.9)	.048
Lymph node or organ metastasis at baseline	Yes	No	112	2.2 (1.2 to 4.1)	.01	110	2.1 (1.2 to 3.8)	.01
Did patient ever have surgery	Yes	No	117	0.3 (0.2 to 0.5)	<.0001	115	0.4 (0.2 to 0.6)	<.0001
Did patient ever receive radiation	Yes	No	117	0.4 (0.3 to 0.6)	<.0001	115	0.5 (0.3 to 0.8)	.004
Did patient ever receive chemotherapy	Yes	No	117	1.1 (0.5 to 2.2)	.8	115	1.4 (0.7 to 2.9)	.4
Microscopic margins involved (in any surgery)	Yes	No	31	10.1 (1.3 to 77.3)	.03	31	15 (2 to 113.8)	.009
Any gross residual disease postoperatively (in any surgery)	Yes	No	47	3.8 (1.4 to 10)	.007	47	3.2 (1.4 to 7.5)	.006
Initial best response	Complete or partial response	Stable or progressive disease	112	0.5 (0.3 to 0.8)	.001	110	0.5 (0.3 to 0.7)	.0002
Bone and soft tissue primary tumor	Yes	No	131	0.5 (0.2 to 1.1)	.07	128	0.7 (0.3 to 1.6)	.4

* CI = confidence interval; EFS = event-free survival; HR = hazard ratio; OS = overall survival; tumor size = largest diameter. BRD = Bromodomain-containing Protein; NUT = Nuclear Protein in Testis; NUTM1 = NUT Midline Carcinoma Family Member 1; NSD = Nuclear Receptor Binding SET Domain Protein.

A recent description of *NUTM1*-rearranged tumors of soft tissue (8) prompted us to examine outcomes in our subset of NC cases arising from soft tissue or bone origin compared with those of typical NC. Interestingly, we found that NCs arising from soft tissue and bone trend toward better OS (HR = 0.5, 95% CI = 0.2 to 1.1, *P = *.07).

### A Proposed Risk Classification Tree for OS

Using outcomes and *NUTM1* fusion identities from this largest cohort of NC patients with available data, we sought to identify clinically and molecularly distinct subsets of NC patients with statistically significantly different prognosis based on OS using survival tree regression (see Supplementary Methods and Supplementary Table 2, available online).

**Figure 2.**
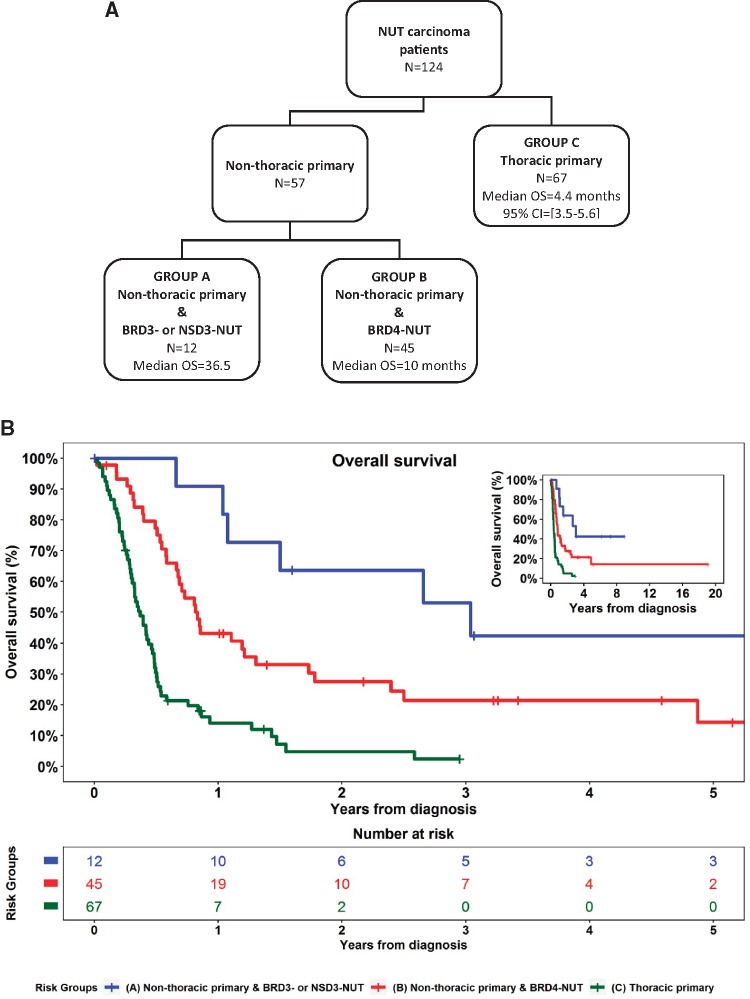
Proposed risk classification tree for overall survival (OS). **A**) Prognostic risk classification model for NUT carcinoma OS outcomes (N* *=* *124). Nonthoracic primary tumors include head and neck, bone, soft tissue, and other sites. **B**) OS for three statistically distinct risk groups truncated at 5 years (N* *=* *124). Inset: OS by risk group across full length of follow-up.

Our analysis (Figure 2, A and B) identified the following three statistically distinct risk groups in 124 patients with available NUT fusion, primary site, and OS data in descending order of OS: group A is nonthoracic, BRD3-NUT, or NSD3-NUT (n = 12, median OS = 36.5 months, 95% CI = 12.5 months to NR); group B is nonthoracic, BRD4-NUT (n = 45, median OS = 10 months, 95% CI = 7 to 14.6 months); and group C is thoracic, regardless of NUT fusion (n = 67, median OS = 4.4 months, 95% CI = 3.5 to 5.6 months). Of note, dichotomizing patients using the strongest effect size (HR) rather than statistical significance (*P* value) produces the same risk classification model. Notably, for the 124 total patients in the risk model, there were five of 12 (42%) long-term survivors (≥3 years) in group A, seven of 45 (16%) in group B, but none in group C (Table 3). Thoracic NC patients (group C) had the poorest OS regardless of the NUT fusion. Group C was remarkable for having 98% disease specific mortality (n* = *50 of 51 with available cause of death and OS = 5%, 95% CI = 1% to 14%) at 2 years. In comparison, 2-year OS was 64% (95% CI = 30% to 85%) in group A and 28% (95% CI = 15% to 42%) in group B (Table 3).

**Table 3.** Patient demographic and clinical characteristics by risk group (N = 124)

Patient characteristic	Group A Nonthoracic primary* and BRD3- or NSD3-NUT	Group B Nonthoracic primary* and BRD4-NUT	Group C Thoracic primary
	(n = 12)	(n = 45)	(n = 67)
Median OS (95% CI), mo	36.5 (12.5 to NR)	10 (7 to 14.6)	4.4 (3.5 to 5.6)
2-y OS (95% CI), %	64 (30 to 85)	28 (15 to 42)	5 (1 to 14)
Long-term survivors (≥3 y), no. (%)	5 of 12 (42)	7 of 45 (16)	0 of 67 (0)
Median age at initial cancer diagnosis (range), y	27.0 (13.4–54.6)	21.9 (0.1–71.4)	23.7 (5.8–68.3)
No.	12	42	59
Age at initial cancer diagnosis, no. (%), y			
<18	5 of 12 (42)	19 of 42 (45)	19 of 59 (32)
≥18	7 of 12 (58)	23 of 42 (55)	40 of 59 (68)
Unknown	0	3	8
Sex, no. (%)			
Male	8 of 12 (67)	19 of 45 (42)	36 of 67 (54)
Female	4 of 12 (33)	26 of 45 (58)	31 of 67 (46)
Primary tumor site, no. (%)			
Thoracic	0 of 12 (0)	0 of 45 (0)	67 of 67 (100)
Head and neck	9 of 12 (75)	40 of 45 (89)	0 of 67 (0)
Bone and soft tissue*	3 of 12 (25)	3 of 45 (7)	0 of 67 (0)
Other site (kidney)	0 of 12 (0)	2 of 45 (4)	0 of 67 (0)
Histological type, no. (%)			
Carcinoma with squamous differentiation	5 of 12 (42)	16 of 43 (37)	21 of 67 (31)
Carcinoma without squamous differentiation	7 of 12 (58)	24 of 43 (56)	36 of 67 (54)
Other histology	0 of 12 (0)	3 of 43 (7)	10 of 67 (15)
Unknown	0	2	0
Tumor diameter at diagnosis, no. (%), cm			
<6	5 of 7 (71)	18 of 27 (67)	18 of 45 (40)
≥6	2 of 7 (29)	9 of 27 (33)	27 of 45 (60)
Unknown	5	18	22
Lymph node or organ metastasis at the baseline, no. (%)			
Yes	4 of 8 (50)	27 of 36 (75)	58 of 62 (94)
No	4 of 8 (50)	9 of 36 (25)	4 of 62 (6)
Unknown	4	9	5
Initial treatment sequence, no. (%)			
Chemotherapy ± subsequent treatment	5 of 10 (50)	9 of 41 (22)	25 of 55 (45)
Radiation ± concurrent chemotherapy ± subsequent treatment	2 of 10 (20)	11 of 41 (27)	18 of 55 (33)
Surgery ± subsequent treatment	3 of 10 (30)	21 of 41 (51)	12 of 55 (22)
None or unknown	2	4	12
Microscopic margins involved (in any surgery), no. (%)†			
Yes	7 of 9 (78)	7 of 12 (58)	6 of 7 (86)
No	2 of 9 (22)	5 of 12 (42)	1 of 7 (14)
Unknown	1	14	10
Any gross residual disease postoperatively (in any surgery), no. (%)†			
Yes	4 of 10 (40)	11 of 19 (58)	11 of 12 (92)
No	6 of 10 (60)	8 of 19 (42)	1 of 12 (8)
Unknown	0	7	5

* Nonthoracic primary tumors include head and neck, bone, soft tissue, and other sites. Bone and soft tissue primary tumor include chest wall soft tissue, soft tissue mass, right hip bone, left scapula or shoulder, right chest soft tissue, soft tissue, occipital scalp soft tissue, iliac bone, temporal region soft tissue mass. CI = confidence interval; OS = overall survival; NR = not reported.

† For patients with surgery and known data.

To examine the potential confounding effect of metastasis on the selected risk factors, we performed post hoc multivariable Cox proportional-hazards regression assessing primary tumor site and gene fusion in the full cohort (Supplementary Table 3, available online). These multivariable models demonstrate that metastasis is no longer an independent predictor of OS after accounting for the effect of tumor site (model 1); gene fusion is a statistically significant predictor of OS independent of tumor site (model 2); and gene fusion remains a statistically significant predictor of OS independent of tumor site, even after controlling for the effects of metastasis (model 3). We further characterized the clinical characteristics and treatment for tumors of thoracic vs nonthoracic origin (Supplementary Table 4, available online). Thoracic tumors were more likely to be associated with metastases at presentation compared with those of nonthoracic origin (94% vs 73%, *P = *.0035) and were larger at presentation (≥6 cm: 60% vs 31%, *P = *.001). Surgery was less likely to ever be performed on patients with thoracic vs nonthoracic NC (30% vs 73%; *P* < .0001).

Demographic and clinical characteristics of the three risk groups differed somewhat (Table 3). BRD3- or NSD3-NUT nonthoracic patients (group A) were less likely to present with metastasis than other groups (50% of patients in group A lacked lymph node or organ metastases vs 25% and 6% for groups B and C, respectively) and had smaller tumors (<6 cm: 71% for group A vs 67% and 40% for groups B and C, respectively). Among patients who received surgery, patients in group A were more likely to have undergone a complete surgical resection with no residual disease (60% for group A vs 42% and 8% for groups B and C, respectively).

### OncoPanel Analysis

Molecular profiling (n* = *10 patients) showed no additional oncogenic mutations of known statistical significance, focal copy number variation, or genomic rearrangements other than the *BRD4-NUTM1* translocation (Supplementary Table 5, available online). The somatic variants present were nonrecurrent. The findings are consistent with previous data indicating that NCs are genetically stable and driven by a single NUT fusion oncoprotein (21); however, findings are not conclusive because of the limited number of samples tested by OncoPanel.

### Treatment and Impact of Therapy

Despite varying outcomes associated with initial treatment, there is no clear-cut difference in treatments that explains the statistically significant difference in outcomes for the three distinct anatomic and genomic prognostic risk groups (Table 3). In fact, initial treatments were similar among two of the three prognostic risk groups (groups A and C; Table 3). Patients in groups A and C were more likely to be treated with up-front chemotherapy, whereas patients in the nonthoracic, BRD4-NUT risk group (group B) were most often treated initially with surgery.

### Minimum Sample Size Needed for Validation Cohort

An independent validation cohort based on future NC patients who prospectively enroll in the NMC registry is necessary to validate the proposed NC risk classification model. Our simulations estimate that a minimum of 198 total patients is required to achieve 80% power to validate the proposed NC risk classification model (Supplementary Table 6, available online). To account for missing data, we expect to accrue up to 210 total patients to have complete data on NUT fusion, primary tumor site, and OS data on the required 198 patients. Note that a validation cohort with a total of 124 patients (eg, the same sample size as the current analysis) only has 56% power to validate the proposed model.

## Discussion

The findings herein, using outcomes and molecular data in this 141 cohort of NC patients, have facilitated the first prognostic risk classification model for NC. The three risk groups reveal that nonthoracic location is associated with a statistically significantly improved prognosis, and nonthoracic NC patients whose tumor has a *BRD3-NUTM1* or *NSD3-NUTM1* fusion are within the best prognostic group. Thoracic NC has a dismal and worst prognosis regardless of the *NUTM1* fusion partner. Of note, within thoracic NC, BRD4-NUTM1 fusion still conferred poorer OS, but the association was not statistically significant (*P* = .09; Supplementary Table 2, available online). Our findings, if validated, indicate that molecular testing to characterize *NUTM1* fusion oncogenes may have important prognostic implications that can help guide treatment decisions. This being said, it is important to appreciate that the type of *NUTM1* fusion may not be the only variable that determines the distinct prognosis for each of the nonthoracic groups (A and B), because these groups include a small number of heterogenous patients; thus, other variables, including differences in treatment and tumor location, may contribute to the differences in outcome in these groups.

NCs that arise within the thorax, in contrast with those that arise elsewhere (head and neck or soft tissue or bone), are thought to present at a more advanced stage and localized growth because of a delay in detection. In our study, thoracic tumors were more likely to be larger (≥6 cm diameter) and to present with metastases compared with nonthoracic tumors; however, multivariable analysis revealed that thoracic site (or gene fusion) is predictive of OS independent of metastasis (Supplementary Table 3, available online). The difference in prognosis may be due to lack of accessibility of thoracic primaries (surgery was performed in 73% of nonthoracic vs 30% of thoracic NC; Supplementary Table 2, available online) and/or possibly a cell of origin with differing biology. The trend of bone and soft tissue primary NCs to have better OS than thoracic or head and neck sites (HR = 0.5, *P = *.07; Table 2), though underpowered because of a small sample size (N* *=* *9), suggests a differing biology and supports the idea that NCs with differing cells of origin may behave differently.

It is intriguing to postulate that the biology of BRD3-NUT- or NSD3-NUT-driven NC may differ somewhat from that of BRD4-NUT tumors, but there are no data to support a substantial difference in the respective molecular pathways. Both BRD3 and NSD3 associate with BRD4 in BRD4-NUT complexes (5,6,22); thus, the components of any NUT fusion complex, regardless of its fusion partner, are predicted to be like those of the BRD4-NUT fusion complex. Indeed, we have previously shown that NSD3-NUT can completely replace the function of BRD4-NUT in NC cells depleted of BRD4-NUT (5). Nevertheless, it is possible that either BRD3-NUT or NSD3-NUT function differs somewhat from that of BRD4-NUT and/or that these fusions target a different cell of origin.

It is possible that the heterogenous behavior of NCs may be due to collaborative mutations. However, the data presented (Supplementary Table 4, available online), in addition to those published (21,23), suggest that clinically relevant oncogenic mutations in addition to the *NUTM1* fusion oncogene are rare or do not occur.

The risk classification model presented indicates better outcomes for nonthoracic and BRD3-NUT or NSD3-NUT NC and has prognostic and treatment implications for physicians and patients. However, our results need to be validated in a prospective cohort before definitive recommendations are made. Assuming an enrollment rate of 25 patients per year in the NMC Registry, we would need approximately 8 years to accrue the required 198 total patients (with complete data) to have 80% power to validate our proposed risk classification model. The fact that all but one of the deceased thoracic patients died of disease, despite varied treatments, implicates a dire prognosis for these patients regardless of stage of disease. Although up-front surgery appears to be effective and curative in some patients with nonthoracic NC (Supplementary Table 2, available online), alternative therapeutic approaches are clearly required for the thoracic NC patients. Currently, new trials with novel bromodomain inhibitors that target BRD3/4 are in development and demonstrate on-target activity in NC (14), with partial responses seen in 20% (24) to 30% (25) of patients treated on phase I trials. However, responses were not durable, in part because of dose-limiting toxicity, and all patients eventually succumbed to disease. Thus, combinatorial approaches are likely to be necessary for this aggressive cancer.

Our study has several limitations. Only 124 of 141 (88%) patients were included in the proposed NC risk classification model. Seventeen patients were excluded because of having 1) unknown OS outcomes, 2) an unknown primary site, 3) missing or unidentified NUT fusions, and/or 4) less frequent *NUTM1* fusions (eg, *ZNF532-NUTM1* and *ZNF592-NUTM1* fusions were excluded). Given the retrospective nature of the registry, response to treatment was determined by physician report rather than by RECIST imaging criteria. Finally, we did not adjust for testing multiple hypotheses. However, we intend to validate our risk classification model in a prospectively collected validation cohort.

Clinical and molecular analysis of 141 NC patients confirms the generally poor outcomes in this disease, but we identify three clinically and molecularly defined prognostic risk groups whose outcomes differ statistically significantly. The analysis provides the most comprehensive understanding of the natural history of NC and highlights the desperate need for effective therapies, particularly for patients with thoracic NC.

### Funding

This work was supported by GlaxoSmithKline (to CAF, NGC, CM, HA, and KD, no grant number is applicable); National Institutes of Health (grant number R01CA124633-10S1) (French).

### Notes

Affiliations of authors: Dana-Farber Cancer Institute, Boston, MA (NGC, CSL, RIH, GIS); Harvard Medical School, Boston, MA (NGC, CM, VN, CSL, SGD, RIH, GIS, SES, CAF); Dana-Farber/Boston Children’s Cancer and Blood Disorders Center, Boston, MA (CM, HA-S, SGD, SES); Brigham and Women’s Hospital, Boston, MA (KD, RB, CAF); Massachusetts General Hospital, Boston, MA (VN); GlaxoSmithKline, Collegeville, PA (AD, JJN, CAF).

CAF, NGC, CM, HA, and KD report receiving research funding from GlaxoSmithKline (GSK) for this project. CAF reports receiving consultant fees from GSK. SGD reports an honorarium from Loxo Oncology and travel expenses from Loxo Oncology and Roche/Genentech. AD and JJN are employees of GSK. All remaining authors report no disclosures.

We thank all patients and their families who enrolled in the NMC Registry, and the clinicians who provided data. We thank GlaxoSmithKline and the National Institutes of Health for funding this work.

The funders had no role in the design of the study; the collection, analysis, and interpretation of the data; the writing of the manuscript; and the decision to submit the manuscript for publication.

## Supplementary Material

pkz094_Supplementary_Data

## References

[1] French CA, Miyoshi I, Kubonishi I, Grier HE, Perez-Atayde AR, Fletcher JA. BRD4-NUT fusion oncogene: a novel mechanism in aggressive carcinoma. Cancer Res. 2003;63(2):304–307. 12543779

[2] French CA, Kutok JL, Faquin WC, et al Midline carcinoma of children and young adults with NUT rearrangement. J Clin Oncol. 2004;22(20):4135–4139. 15483023 10.1200/JCO.2004.02.107

[3] Bauer DE, Mitchell CM, Strait KM, et al Clinicopathologic features and long-term outcomes of NUT midline carcinoma. Clin Cancer Res. 2012;18(20):5773–5779. 22896655 10.1158/1078-0432.CCR-12-1153 PMC3473162

[4] French CA, Ramirez CL, Kolmakova J, et al BRD-NUT oncoproteins: a family of closely related nuclear proteins that block epithelial differentiation and maintain the growth of carcinoma cells. Oncogene. 2008;27(15):2237–2242. 17934517 10.1038/sj.onc.1210852

[5] French CA, Rahman S, Walsh EM, et al NSD3-NUT fusion oncoprotein in NUT midline carcinoma: implications for a novel oncogenic mechanism. Cancer Discov. 2014;4(8):928–941. 24875858 10.1158/2159-8290.CD-14-0014 PMC4125436

[6] Alekseyenko AA, Walsh EM, Zee BM, et al Ectopic protein interactions within BRD4 chromatin complexes drive oncogenic megadomain formation in NUT midline carcinoma. Proc Natl Acad Sci USA. 2017;114(21):E4184–E4192. 28484033 10.1073/pnas.1702086114 PMC5448232

[7] Schaefer IM, Dal Cin P, Fletcher CDM, Hanna GJ, French CA. CIC-NUTM1 fusion: a case which expands the spectrum of NUT-rearranged epithelioid malignancies. Genes Chromosomes Cancer. 2018;57(9):446–451. 29700887 10.1002/gcc.3 PMC6881821

[8] Dickson BC, Sung YS, Rosenblum MK, et al NUTM1 gene fusions characterize a subset of undifferentiated soft tissue and visceral tumors. Am J Surg Pathol. 2018;42(5):636–645. 29356724 10.1097/PAS.0000000000001021 PMC5893407

[9] Shiota HE, Danga K, Becht K, et al . Novel ZNF-fusions in NUT carcinoma. Cancer Res. 2018;78(suppl 13):Abstract no. 2487. 10.1158/1541-7786.MCR-18-0474 PMC6279489 30139738

[10] Grayson AR, Walsh EM, Cameron MJ, et al MYC, a downstream target of BRD-NUT, is necessary and sufficient for the blockade of differentiation in NUT midline carcinoma. Oncogene. 2014;33(13):1736–1742. 23604113 10.1038/onc.2013.126 PMC3942361

[11] Alekseyenko AA, Walsh EM, Wang X, et al The oncogenic BRD4-NUT chromatin regulator drives aberrant transcription within large topological domains. Genes Dev. 2015;29(14):1507–1523. 26220994 10.1101/gad.267583.115 PMC4526735

[12] Chau NM, Aserlind A, Grunfeld N, et al Aggressive treatment and survival outcomes in NUT midline carcinoma (NMC) of the head and neck (HN). J Clin Oncol. 2014;32(suppl 15).

[13] Mertens F, Wiebe T, Adlercreutz C, Mandahl N, French CA. Successful treatment of a child with t(15; 19)-positive tumor. Pediatr Blood Cancer. 2007;49(7):1015–1017. 16435379 10.1002/pbc.20755

[14] Stathis A, Zucca E, Bekradda M, et al Clinical response of carcinomas harboring the BRD4-NUT oncoprotein to the targeted bromodomain inhibitor OTX015/MK-8628. Cancer Discov. 2016;6(5):492–500. 26976114 10.1158/2159-8290.CD-15-1335 PMC4854801

[15] Haack H, Johnson LA, Fry CJ, et al Diagnosis of NUT midline carcinoma using a NUT-specific monoclonal antibody. Am J Surg Pathol. 2009;33(7):984–991. 19363441 10.1097/PAS.0b013e318198d666 PMC2783402

[16] French CA, den Bakker MA. NUT carcinoma In: WD Travis, E Brambilla, AP Burke, Marx A, Nicholson AG, eds. *WHO Classification of Head and Neck Tumours* 4th ed. Lyon, France: International Agency for Research on Cancer (IARC); 2015:412.

[17] Chirieac LR, French CA, Sholl L, Yatabi Y. Lung: other and unclassified carcinomas In: WD Travis, E Brambilla, Burke AP, A Marx, AG Nicholson, eds. *WHO Classification of Tumours of the Lung, Pleura, Thymus and Heart* 4 ed. Herndon, VA: Stylus Publishing, LLC; 2015:2.

[18] Vargas SO, French CA, Faul PN, et al Upper respiratory tract carcinoma with chromosomal translocation 15; 19: evidence for a distinct disease entity of young patients with a rapidly fatal course. Cancer. 2001;92(5):1195–1203. 11571733 10.1002/1097-0142(20010901)92:5<1195::aid-cncr1438>3.0.co;2-3

[19] Segal MR. Regression trees for censored data. Biometrics. 1988;44(1):35–47.

[20] Cohn SL, Pearson AD, London WB, et al The International Neuroblastoma Risk Group (INRG) classification system: an INRG task force report. J Clin Oncol. 2009;27(2):289–297. 19047291 10.1200/JCO.2008.16.6785 PMC2650388

[21] Lee JK, Louzada S, An Y, et al Complex chromosomal rearrangements by single catastrophic pathogenesis in NUT midline carcinoma. Ann Oncol. 2017;28(4):890–897. 28203693 10.1093/annonc/mdw686 PMC5378225

[22] Rahman S, Sowa ME, Ottinger M, et al The Brd4 extraterminal domain confers transcription activation independent of pTEFb by recruiting multiple proteins, including NSD3. Mol Cell Biol. 2011;31(13):2641–2652. 21555454 10.1128/MCB.01341-10 PMC3133372

[23] Stirnweiss A, Oommen J, Kotecha RS, Kees UR, Beesley AH. Molecular-genetic profiling and high-throughput in vitro drug screening in NUT midline carcinoma-an aggressive and fatal disease. Oncotarget. 2017;8(68):112313–112329. 29348827 10.18632/oncotarget.22862 PMC5762512

[24] O’Dwyer PP-P, French CA, Harward S, et al Abstract CT014: GSK525762, a selective bromodomain (BRD) and extra terminal protein (BET) inhibitor: results from part 1 of a phase I/II open-label single-agent study in patients with NUT midline carcinoma (NMC) and other cancers. Cancer Res. 2016;76(suppl 14).

[25] Lewin J, Soria JC, Stathis A, et al Phase Ib trial with birabresib, a small-molecule inhibitor of bromodomain and extraterminal proteins, in patients with selected advanced solid tumors. J Clin Oncol. 2018;36(30):3007–3014. 29733771 10.1200/JCO.2018.78.2292

