# Supplementary material for: An Anatomical Site and Genetic-Based Prognostic Model for Patients With Nuclear Protein in Testis (NUT) Midline Carcinoma: Analysis of 124 Patients
Source: JNCI Cancer Spectr. 2019 Nov 6;4(2):pkz094. doi: 10.1093/jncics/pkz094 (PMC7165803; doi:10.1093/jncics/pkz094)
Supplement: pkz094_Supplementary_Data [file pkz094_supplementary_data.pdf]

Supplemental Methods

International NUT Midline Carcinoma Registry

In 2010, the International NMC Registry was created to serve as a central repository to capture clinical, genetic, pathologic characteristics, and outcomes of NC patients covering pts diagnosed from 1993 to present. The NMC Registry is international, and as such includes patients from North and South America, Europe, Asia, Australia, and New Zealand, however the majority of these patients were from the U.S. The criterion for enrollment was a diagnosis of NC. Patients were enrolled after signing the Institutional Review Board (IRB)-approved informed consent, whereas there was an IRB-approved waiver of consent for deceased patients thereby allowing retrospective entry into the registry.

Patients

Initial treatment was defined as the first treatment given after the initial diagnosis of cancer (which may not have been NC) to the first relapse or progression. The extent of surgery was categorized as complete resection with negative margins (R0 resection), gross total resection (resection of all gross visible disease but with microscopic residual disease), or debulking (gross residual disease present). Clinical responses to initial treatment interventions were classified as progressive disease (PD), stable disease (SD), partial response (PR), or complete response (CR) based on the clinical judgment of the treating physician or Registry oncologist (NC), upon review of the medical record.

NUT Immunohistochemistry

IHC for NUT was performed on 5-micron-thick, formalin-fixed, paraffin-embedded sections (FFPE) using primary rabbit monoclonal anti-NUT (clone C52B1,1:50) (Cell Signaling Technology, Danvers, MA) as previously described[1].

FISH

Dual color bring-together and split-apart FISH on 5 micron FFPE sections of tumor was performed as described[2]. FISH probes used for characterizing the *NUTM1*-fusion were as following: *NUTM1*: 5' centromeric probes, RP11-368L15 and RP11-1084A12 (biotin labeled, red) and 3' telomeric probes, RP11-1H8 and RP11-64o3 (digoxigenin labeled, green); *BRD4*: 5' centromeric probes, RP11-207i16 and RP11-3055m5 (biotin labeled, red), and 3' telomeric probes, RP11-319O10 and RP11-681D10 (digoxigenin labeled, green); *NSD3*: 5′ centromeric BAC clones CTD-2538P2 and RP11-957P17 (biotin labeled, red) and the 3′ telomeric BAC clones CTB-497A2 and RP11-90P5 (digoxigenin labeled, green); *BRD3*: 5' telomeric probes, RP11-145E17 and RP11-92B21 (biotin labeled, red), and 3' centromeric probes, RP11-260C10 and RP11-153P4 (digoxigenin labeled, green); *ZNF532*: 3′ telomeric BAC probes RP11-350K6 and -1061A13 (digoxigenin labeled, green) and the 5′ centromeric probes RP11-351N16 and -722P5 (biotin labeled, red). 200 nuclei were counted in four different areas of each tumor. Eighty percent positive interpretable nuclei were defined as positive for a rearrangement.

Archer® FusionPlex®

Archer was performed in the Center for Integrated Diagnostics (CID) in the department of pathology at the Massachusetts General Hospital for select cases where the fusion partner to *NUTM1* was not identified by FISH or cytogenetics. An Anchored Multiplex PCR (AMP) assay was performed with Archer® FusionPlex® Solid Tumor Kit (ArcherDX, Boulder, CO) for detection of targeted fusion transcripts using next generation sequencing (NGS)[3]. Total nucleic acid was isolated from FFPE sections after histological review for tumor enrichment. The total nucleic acid was reverse transcribed with random hexamers, followed by second strand synthesis

to create double-stranded complementary DNA (cDNA). The double-stranded cDNA was end-repaired, adenylated, and ligated with a half-functional adapter. Two hemi-nested PCR reactions using the Archer® FusionPlex® Solid Tumor Kit primers were performed to create a fully functional sequencing library that targets specific genes (exons) listed previously[4] and validated for clinical reporting, including amongst multiple other genes *BRD3* (exons 9-12), *BRD4* (exons 10, 11), and *NUTM1* (exon 3). Illumina NextSeq 2 x 150 base paired-end sequencing results were aligned to the hg19 human genome reference using bwa-mem[5]. A laboratory-developed algorithm was used for fusion transcript detection and annotation[4]. The integrity of the input nucleic acid and the technical performance of the assay were assessed with a qualitative reverse transcription qPCR assay and assessing the DNA/RNA content in the sequencing results. The assay is validated for samples showing 5% or higher tumor cellularity.

Next-generation (OncoPanel) targeted sequencing

OncoPanel molecular profiling of FFPE sections of tumors was performed in patients who received routine care at the Dana-Farber Cancer Institute (DFCI) and had consented to an IRB-approved, institute-wide research protocol. DNA extracted from FFPE tissue was subjected to targeted exon hybrid capture (Agilent, Santa Clara, CA) and NGS using an Illumina HiSeq 2500 (Illumina, San Diego, CA). Exons of 447 cancer-associated genes were interrogated for mutations and copy number variations, and 191 introns across 60 genes were examined for structural rearrangements, using the targeted sequencing platform developed at Brigham and Women's Hospital (BWH[6]). Bioinformatic detection of single nucleotide variants and small indels was performed using MuTect and GATK software. RobustCNV (an internally developed tool) was used for copy number analysis, and BreaKmer for large structural variations as described[7, 8].

Simulation study to determine the sample size needed to validate the risk classification model

We performed computer simulations to determine the minimum sample size of a prospectively-collected NC patient cohort to validate our proposed risk classification model with 80% power. We used the observed characteristics of the prognostic factors and overall survival outcomes in our current cohort of N=141 patients to simulate "mock" validation datasets. We then reconstructed the proposed risk classification model using the simulated dataset: we first dichotomized patients by primary tumor site, and then further dichotomized non-thoracic patients by NUT translocation. We performed Cox proportional-hazards regression at each of the two "splits" in the survival tree. If both Cox models were statistically significant ($p<0.05$), we declared our risk classification model "validated" in this simulated dataset. Conversely, if one or more Cox model was not statistically significant, we declared our risk classification model not validated in this simulated dataset. We then repeated this process in 10,000 independently simulated datasets. The power to validate the risk classification model is the proportion of 10,000 simulation replicates where the risk classification model was validated. We assessed the validation power across 18 different sample sizes for the validation cohort, ranging from N=100 to 200.

Details for generating the risk classification model using survival tree regression

Using survival tree regression analysis, we selected the risk factor with the strongest statistical association (e.g. lowest p-value) to recursively dichotomize the patients into risk groups. We first dichotomized patients by primary tumor site, which was the risk factor with the strongest univariate association in the full cohort (HR=3.4 [95% CI=2.2-5.1]; $p<0.0001$; Table 2). Within the subset of patients with thoracic primaries (N=67), none of the remaining risk factors were significantly associated with OS ($p>0.07$; Supplementary Table S1); thus, the thoracic patient

subset was not further dichotomized. For the subset of patients with non-thoracic primaries (N=64), we further dichotomized patients by gene fusion, which was the only significant risk factor in this subset (HR=2.5 [95%CI=1-6]; p=0.042). Since no further risk factors were significant within the gene fusion subsets, patients were not further dichotomized.

Supplementary Table S1: Country of origin for NUT midline cases (N=141)

Country	Frequency	Percent
USA	92	65.24
Unknown	14	9.92
Australia	6	4.25
Italy	6	4.25
Canada	3	2.13
Netherlands	3	2.13
Sweden	3	2.13
Ireland	2	1.42
Japan	2	1.42
Switzerland	2	1.42
Brazil	1	0.71
China	1	0.71
Croatia	1	0.71
France	1	0.71
Germany	1	0.71
Greece	1	0.71
New Zealand	1	0.71
Palestine	1	0.71

Supplementary Table S2: Univariate Cox proportional-hazards regression for NUT carcinoma risk classification subsets

	Level	Reference	N	Hazard Ratio (95% CI*)	p-value
Thoracic subset, N=67					
Age at diagnosis (Yr)	≥18 years	<18 years	59	1.2 (0.7-2.2)	0.5
Sex	Female	Male	67	1 (0.6-1.6)	0.9
Histopathologic type of tumor	Carcinoma without squamous differentiation & other histopathology	Carcinoma with squamous differentiation	67	1.2 (0.7-2.1)	0.5
Tumor size	≥6 cm	<6 cm	45	1.2 (0.6-2.3)	0.6
Gene fusion	BRD4-NUTM1	BRD3-NUTM1 or NSD3-NUTM1	59	1.8 (0.9-3.7)	0.09
Lymph node/Organ Metastasis at the baseline	Yes	No	62	1.6 (0.6-4.5)	0.4
Bone/Soft Tissue Primary Tumor**	NA	NA	NA	NA	NA
Non-thoracic subset, N=64					
Age at diagnosis (Yr)	≥18 years	<18 years	61	1.7 (0.9-3.2)	0.1
Sex	Female	Male	64	1.2 (0.6-2.1)	0.6
Histopathologic type of tumor	Carcinoma without squamous differentiation & other histopathology	Carcinoma with squamous differentiation	61	1.0 (0.6-1.9)	0.9
Tumor size	≥6 cm	<6 cm	38	1.2 (0.5-2.7)	0.7
Gene fusion	BRD4-NUTM1	BRD3-NUTM1 or NSD3-NUTM1	57	2.5 (1-6)	0.042
Lymph node/Organ Metastasis at the baseline	Yes	No	49	1.5 (0.7-3.4)	0.3
Bone/Soft Tissue Primary Tumor	Yes	No	64	0.7 (0.3-1.7)	0.5
Non-thoracic BRD4-NUT subset, N=45					
Age at diagnosis (Yr)	≥18 years	<18 years	42	1.6 (0.8-3.3)	0.2
Sex	Female	Male	45	1.2 (0.6-2.5)	0.5
Histopathologic type of tumor	Carcinoma without squamous differentiation & other histopathology	Carcinoma with squamous differentiation	43	1.3 (0.6-2.7)	0.5
Tumor size	≥6 cm	<6 cm	27	1 (0.4-2.5)	0.98
Lymph node/Organ Metastasis at the baseline	Yes	No	36	1.1 (0.4-2.8)	0.8
Bone/Soft Tissue Primary Tumor	Yes	No	45	0.6 (0.1-2.5)	0.5

Non-thoracic BRD3-NUT subset, N=12					
No models were run for subsets with <20 patients	NA	NA	NA	NA	NA

*Abbreviations: CI: confidence interval, NA: not applicable, Yr: year

**None of the thoracic patients had bone/soft tissue primary tumor

Supplementary Table S3: Multivariable Cox Proportional Hazards Regression models for overall survival in the full cohort

Risk Factor	Level	Reference	Adjusted Hazard Ratio (95% CI*)	Adjusted p-value
Model 1 (N=116)				
Primary tumor site	Thoracic	Non-thoracic (head and neck, bone, soft tissue, & other)	3.8 (2.3-6.1)	<0.0001
Lymph node/Organ Metastasis at the baseline	Yes	No	1.5 (0.8-2.9)	0.18
Model 2 (N=111)				
Primary tumor site	Thoracic	Non-thoracic (head and neck, bone, soft tissue, & other)	3.3 (2.2-5.2)	<0.0001
Gene fusion	BRD4-NUTM1	BRD3-NUTM1 or NSD3-NUTM1	2.1 (1.2-3.5)	0.008
Model 3 (N=99)				
Primary tumor site	Thoracic	Non-thoracic (head and neck, bone, soft tissue, & other)	3.7 (2.2-6.1)	<0.0001
Lymph node/Organ Metastasis at the baseline	Yes	No	1.4 (0.7-2.7)	0.29
Gene fusion	BRD4-NUTM1	BRD3-NUTM1 or NSD3-NUTM1	1.8 (1-3.2)	0.047

Supplementary Table S4: Clinical characteristics and treatment between thoracic and non-thoracic primary tumors [N=140*]

Patient Characteristic	Category	Non-thoracic primary N=69	Thoracic primary N=71	Fisher's exact test p-value
Lymph node and/or organ metastasis at presentation	Yes	37/51 (73)	60/64 (94)	0.0035
	No	14/51 (27)	4/64 (6)	
Tumor size at diagnosis (diameter)	<6 cm	27/39 (69)	19/47 (40)	0.001
	>=6 cm	12/39 (31)	28/47 (60)	
Did the patient have surgery at any time?	Yes	41/56 (73)	18/61 (30)	<0.0001
	No	15/56 (27)	43/61 (70)	

* Excludes one patient with unknown primary site.

Supplementary Table S5: Genomic alterations identified by Oncopanel

Single nucleotide variants								
Patient ID	Gene	Exon	DNA change	Protein	Variant Type	Variant allele	Tumor purity	Predicted effect
1	MUTYH	1	c.29G>C	p.R10P	Missense	48% of 50 reads	30%	Unknown
1	GLI2	5	c.764C>T	p.S255L	Missense	48% of 144 reads	30%	Unknown
2	PIK3C2B	30	c.4322C>T	p.A1441V	Missense	5% of 81 reads	90%	Unknown
2	MDM4	11	c.1028C>T	p.T343M	Missense	49% of 235 reads	90%	Unknown
3	EP300	4	c.937C>G	p.P313A	Missense	45% of 134 reads	50%	Unknown
4	ID3	2	c.337G>A	p.D113N	Missense	68% of 119 reads	90%	Unknown
4	CRTC2	6	c.535G>A	p.V179M	Missense	28% of 323 reads	90%	Unknown
4	CTNNB1	10	c.1511G>A	p.W504*	Nonsense	4% of 323 reads	90%	Unknown
4	SF3B1	7	c.788C>T	p.A263V	Missense	52% of 194 reads	90%	Unknown
5	CREBBP	2	c.508C>T	p.Q170*	Nonsense	46% of 408 reads	80%	Unknown
5	GSTM5	7	c.519G>C	p.K173N	Missense	46% of 559 reads	80%	Unknown
5	CSF1R	3	c.172T>C	p.W58R	Missense	49% of 296 reads	80%	Unknown
5	KIT	14	c.2056C>T	p.R686C	Missense	48% of 327 reads	80%	Unknown
5	EP300	1	c.2T>G	p.M1R	Missense	49% of 213 reads	80%	Unknown
6	EPHA7	15	c.2722A>G	p.S908G	Missense	50% of 767 reads	40%	Unknown
7	no somatic mutations were detected							
8	ALK	29	c.4801G>A	p.A1601T	Missense	52% of 251 reads	40%	Unknown
8	BRIP1	7	c.845C>G	p.T282S	Missense	48% of 469 reads	40%	Unknown
8	DEPDC5	41	c.4414A>G	p.I1472V	Missense	49% of 191 reads	40%	Unknown
8	DICER1	3	c.25C>T	p.L9F	Missense	47% of 221 reads	40%	Unknown
8	GNB2L1	--	c.889-162G>A	------------------	Missense	41% of 240 reads	40%	Unknown
8	KMT2D	34	c.8573C>G	p.S2858C	Missense	52% of 249 reads	40%	Unknown
8	NTRK3	19	c.2465A>G	p.K822R	Missense	32% of 223 reads	40%	Unknown
9	no somatic mutations were detected							
10	DICER1	3		p.G258V	Missense			Unknown
Copy number alterations								
Patient ID								
1-10	No focal copy number changes identified.							
Rearrangement analysis								
Patient ID	Gene	Chr.	DNA	Break-point 1	Break-point 2	Variant type		Predicted effect
1-5, 7-9	None	N/A	N/A	N/A	N/A	N/A		N/A
6	BRD4, NUTM1	15, 19	t(15; 19; q14;p13.1)(hg19 chr15:34632617, chr19:15359988)	repeated masked region of the genome 5' to NUTM1 on chr.15	Intron 11 of BRD4	Translocation		BRD4-NUT fusion
10	BRD4, NUTM1	15, 19	t(15; 19; q14;p13.1)(hg19 chr15:34634192, chr19:15351022)	<1.5kb from the 5'UTR of NUTM1 on chr. 15	Intron 14 of BRD4	Translocation		BRD4-NUT fusion

Supplementary Table S6: Simulated power to validate the proposed NUT carcinoma risk classification model for different validation sample sizes.

Sample size in validation cohort	Power to validate NUT carcinoma risk classification model
100	46.15%
110	50.45%
120	55.02%
124	56.16%
130	58.98%
140	63.30%
150	65.65%
160	70.65%
170	72.68%
180	76.53%
190	77.87%
195	78.99%
196	79.78%
197	79.46%
198*	80.16%
199	80.74%
200	80.59%
201	80.51%
202	81.46%
203	80.00%
204	80.50%
205	80.70%
210	82.29%
220	83.84%

*A minimum of 198 patients is required to achieve 80% power to validate the proposed NUT carcinoma risk classification model.

References

1. Stathis A, Zucca E, Bekradda M, Gomez-Roca C, Delord JP, de La Motte Rouge T, et al. Clinical Response of Carcinomas Harboring the BRD4-NUT Oncoprotein to the Targeted Bromodomain Inhibitor OTX015/MK-8628. Cancer discovery. 2016;6(5):492-500.
2. French CA, Kutok JL, Faquin WC, Toretsky JA, Antonescu CR, Griffin CA, et al. Midline carcinoma of children and young adults with NUT rearrangement. J Clin Oncol. 2004;22(20):4135-9.
3. Zheng Z, Liebers M, Zhelyazkova B, Cao Y, Panditi D, Lynch KD, et al. Anchored multiplex PCR for targeted next-generation sequencing. Nature medicine. 2014;20(12):1479-84.

4. Matissek KJ, Onozato ML, Sun S, Zheng Z, Schultz A, Lee J, et al. Expressed Gene Fusions as Frequent Drivers of Poor Outcomes in Hormone Receptor-Positive Breast Cancer. Cancer discovery. 2018;8(3):336-53.
5. Li H, Durbin R. Fast and accurate short read alignment with Burrows-Wheeler transform. Bioinformatics. 2009;25(14):1754-60.
6. Schaefer IM, Dal Cin P, Fletcher CDM, Hanna GJ, French CA. CIC-NUTM1 Fusion: A Case Which Expands the Spectrum of NUT-Rearranged Epithelioid Malignancies. Genes, chromosomes & cancer. 2018.
7. Abo RP, Ducar M, Garcia EP, Thorner AR, Rojas-Rudilla V, Lin L, et al. BreaKmer: detection of structural variation in targeted massively parallel sequencing data using kmers. Nucleic acids research. 2015;43(3):e19.
8. Wagle N, Berger MF, Davis MJ, Blumenstiel B, Defelice M, Pochanard P, et al. High-throughput detection of actionable genomic alterations in clinical tumor samples by targeted, massively parallel sequencing. Cancer discovery. 2012;2(1):82-93.
